# Quantitating intracellular oxygen tension *in vivo* by phosphorescence lifetime measurement

**DOI:** 10.1038/srep17838

**Published:** 2015-12-08

**Authors:** Yosuke Hirakawa, Toshitada Yoshihara, Mako Kamiya, Imari Mimura, Daichi Fujikura, Tsuyoshi Masuda, Ryohei Kikuchi, Ippei Takahashi, Yasuteru Urano, Seiji Tobita, Masaomi Nangaku

**Affiliations:** 1 Graduate School of Medicine, The University of Tokyo, 7-3-1 Hongo, Bunkyo-ku, Tokyo 113-0033, Japan; 2 Graduate School of Science and Technology, Gunma University, 1-5-1 Tenjincho, Kiryu, Gunma 376-8515, Japan; 3 PRESTO, Japan Science and Technology Agency, 4-1-8 Honcho, Kawaguchi, Saitama 332-0012, Japan; 4 Graduate School of Pharmaceutical Sciences, The University of Tokyo, 7-3-1 Hongo, Bunkyo-ku, Tokyo 113-0033, Japan; 5 CREST, Agency for Medical Research and Development, 1-7-1, Otemachi, Chiyoda-ku, Tokyo 100-0004, Japan

## Abstract

Hypoxia appears to have an important role in pathological conditions in many organs such as kidney; however, a method to quantify intracellular oxygen tension *in vivo* has not been well established. In this study, we established an optical method to quantify oxygen tension in mice kidneys using a cationic lipophilic phosphorescence probe, BTPDM1, which has an intracellular oxygen concentration-sensitive phosphorescence lifetime. Since this probe is distributed inside the tubular cells of the mice kidney, we succeeded in detecting acute renal hypoxic conditions and chronic kidney disease. This technique enabled us to estimate intracellular partial pressures of oxygen *in vivo* by extrapolating the calibration curve generated from cultured tubular cells. Since intracellular oxygen tension is directly related to cellular hypoxic reactions, such as the activation of hypoxia-inducible factors, our method will shed new light on hypoxia research *in vivo*.

Hypoxia affects various pathological conditions in many diseases, such as cancer, atherosclerosis, fibrosis, and organ transplant rejection^1^,^2^. The main regulator of cellular reactions to hypoxia is considered to be hypoxia-inducible factor (HIF), a transcriptional activator, which is regulated by intracellular oxygen concentration. Hypoxia plays a crucial role in kidney pathogenesis^3^,^4^,^5^. For example, in the progression of chronic kidney disease (CKD), which constitutes a major public health problem, hypoxia in the tubulointerstitium is the final common pathway^4^. Nevertheless, the detailed mechanism of when and how hypoxia starts to affect the clinical course of CKD remains unclear. One of the reasons for this would be the difficulties encountered during quantitative assessment of intracellular oxygen tension using conventional techniques for detecting hypoxia. Immunohistochemistry of pimonidazole protein adducts and HIF activation reflects intracellular oxygen tension but they are neither quantitative nor indicative of oxygen tension. On the other hand, oxygen electrodes and blood oxygen level dependent-magnetic resonance imaging (BOLD-MRI) enables quantitative assessment, but the obtained values reflect extracellular oxygen tension, which is found in the blood stream^5^,^6^. Phosphorescence lifetime measurement has also been reported to be a useful method for quantitatively monitoring oxygen concentrations *in vivo*^7^,^8^,^9^,^10^,^11^, since phosphorescence lifetime varies with oxygen concentration without the confounding effect of autofluorescence, probe concentration, and reabsorption of phosphorescence mainly by hemoglobin. However, as most of the existing phosphorescence probes tend to distribute in extracellular fluid, we could not perform a direct estimation of intracellular oxygen tension in kidney. One successful example is protoporphyrin IX (PpIX), which is reported to be a useful hypoxia detector with intracellular distribution^12^,^13^, but it takes several hours to produce sufficient amounts of PpIX after application of its precursor 5-aminolevurinic acid (5-ALA). Probably due to this limitation, fluorescence lifetime measurements using 5-ALA have been applied to superficial tissues such as abdominal skin, but not to the intra-abdominal organs. Similarly, most phosphorescence dyes used in the living murine models have some limitations for hypoxia detection and intracellular oxygen tension assessment in the intra-abdominal organs, such as kidney.

Recently, we have reported that BTPDM1, a phosphorescence dye based on the iridium(III) complex BTP, (btp)_2_Ir(acac) (btp = benzothienylpyridine, acac = acetylacetone) with a cationic dimethylamino group, distributes well intracellularly; thus, reporting tumor hypoxia *in vivo* through phosphorescence lifetime measurement is possible^14^. Therefore, we thought that BTPDM1 should also be applicable for quantitatively assessing intracellular oxygen in kidney, when considering its predominant biodistribution to kidney together with its intracellular distribution. In this study, we report quantitative assessment of intracellular oxygen tension in mice kidneys *in vivo* through systemic administration of BTPDM1 followed by phosphorescence lifetime measurements.

## Results

### Distribution and phosphorescence characteristics of BTPDM1 in proximal tubular cells

**Figure 1:**
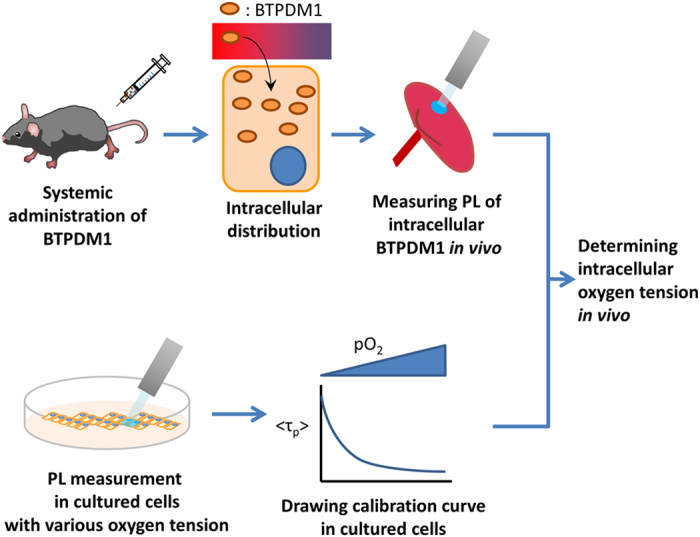
Schematic overview of this study. Our phosphorescence probe, BTPDM1, distributes intracellularly after systemic administration to murine. Thus phosphorescence lifetime of kidney surface correlates to intracellular oxygen tension. To convert phosphorescence lifetime to partial pressure of oxygen, we measured phosphorescence lifetime in cultured cells and generated a calibration curve. By extrapolating this curve to phosphorescence lifetime *in vivo*, we can determine intracellular oxygen tension *in vivo*. Abbreviation; PL, phosphorescence lifetime.

**Figure 2:**
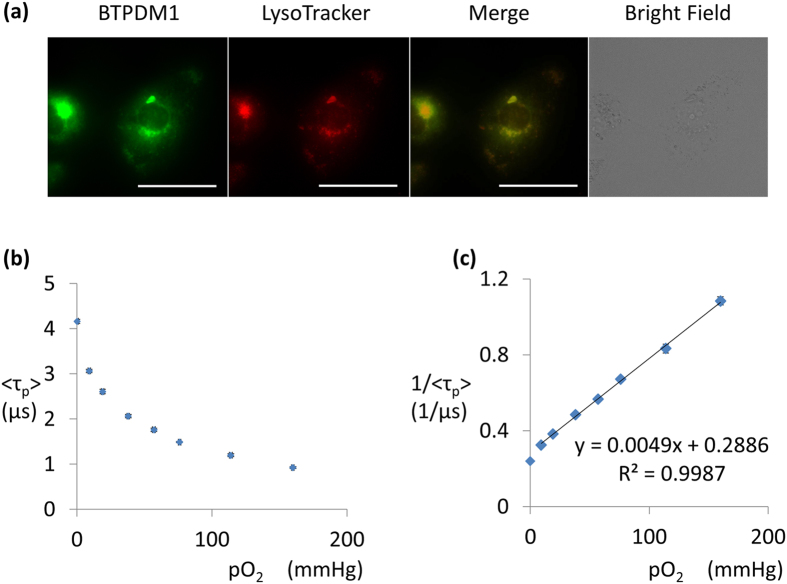
BTPDM1 characteristics in HK-2 cells. (**a**) Phosphorescence/fluorescence image of BTPDM1, LysoTracker, merged image and bright field image of HK-2 cells. The brightness and contrast were adjusted from original in a bright field image. Scale bar: 50 μm. (**b**) Phosphorescence lifetime of BTPDM1 in HK-2 cells in various *p*O_2_ of incubating atmosphere at 37 °C. Each phosphorescence lifetime is the average of four measurements. Error bar: S.D. (**c**) Reciprocal plot of phosphorescence lifetime and oxygen concentration. The approximate line was made by a straight-line approximation and an approximation formula, and the coefficient of determination were also shown.

An overview of this study is shown in Fig. 1. The chemical structure and absorption/phosphorescence spectrum of BTPDM1 are shown in Supplementary Fig. 1a,b. Since the distribution and phosphorescence lifetime of BTPDM1 can vary depending on the cell type, we first confirmed its intracellular distribution and the oxygen dependency of phosphorescence lifetime in HK-2 (human kidney 2) cells. As a result, signals from BTPDM1 colocalized with those from LysoTracker, indicating that BTPDM1 distributed mainly in the lysosomes in HK-2 cells as was the case with other cell types^14^ (Fig. 2a). To prepare a new calibration curve specific for renal tubular cells, we measured phosphorescence lifetime 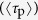 of BTPDM1 in HK-2 cells incubated under various oxygen concentrations according to our previous report^14^. As expected, the phosphorescence lifetime elongated as incubating oxygen concentrations decreased (Fig. 2b).

Given that the decay curve of phosphorescence is described as single-exponential decay, the Stern–Volmer equation holds (1), and thus the reciprocal of the lifetime (τ_p_) and partial pressure of oxygen (pO_2_) should have a linear correlation:


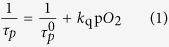


Where 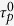 is the phosphorescence lifetime under anoxic condition and *k*_q_ is the bimolecular quenching rate constant. We examined the reciprocal of phosphorescence lifetime according to the Stern–Volmer equation, although not strictly applicable to average lifetime, because this time we used 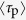, the average lifetime for the observed double-exponential decay curves (see Methods for details), as the lifetime of the cell. As a result, the reciprocal of the phosphorescence lifetimes fitted well with linear approximation by *p*O_2_, except for the lifetime obtained under anoxia conditions (Fig. 2c). This was feasible because cells could not maintain their ordinary function under anoxic conditions, leading to subsequent changes in the distribution of BTPDM1. Therefore, we generated the calibration curve for an oxygen concentration more than 1.2% (9.1 mmHg). The slope was quite different from the calibration line for BTPDM1 in dimyristoylphosphatidylcholine (DMPC) membranes which can be an imitation of intracellular phospholipid membrane^14^. The slope and the intercept of this calibration line were also different from those in squamous cell carcinoma (SCC-7) cells^14^. This demonstrated the importance of generating specific calibration curves with the cells of interest. We also measured the phosphorescence lifetime in HK-2 cells under various oxygen concentrations at 30 °C (Supplementary Fig. 2) because the surface temperature of the kidney for *in vivo* measurements was between 30 °C and 35 °C as described below. We tabulated phosphorescence lifetimes in 0.1 μs increments corresponding to oxygen concentrations at 30 °C and 37 °C, and found that phosphorescence lifetimes were equivalent at the two temperatures (Supplementary table 1). This result suggested that the application of the calibration curve at 37 °C to the result of phosphorescence lifetime measurement *in vivo* is acceptable. We also measured phosphorescence lifetimes under several oxygen concentrations in human primary renal tubular cells (RPTEC) and confirmed that the phosphorescence lifetime did not differ from those in HK-2 cells (Supplementary Fig. 3). This result should justify the use of a calibration curve in HK-2 cells, an immortalized cell line, to convert phosphorescence lifetimes *in vivo* to oxygen tension.

We also investigated the probe toxicity in HK-2 cells. Cell viability did not change with probe concentrations up to 500 nM, which is the probe concentrations in the experiments described above (Supplementary Fig. 4). This result ensured that our probe was not toxic, thus excluding any possible contributions of cell toxicity of the probe to phosphorescence lifetime.

### BTPDM1 distribution and phosphorescence lifetime measurement in normal mice kidneys

**Figure 3:**
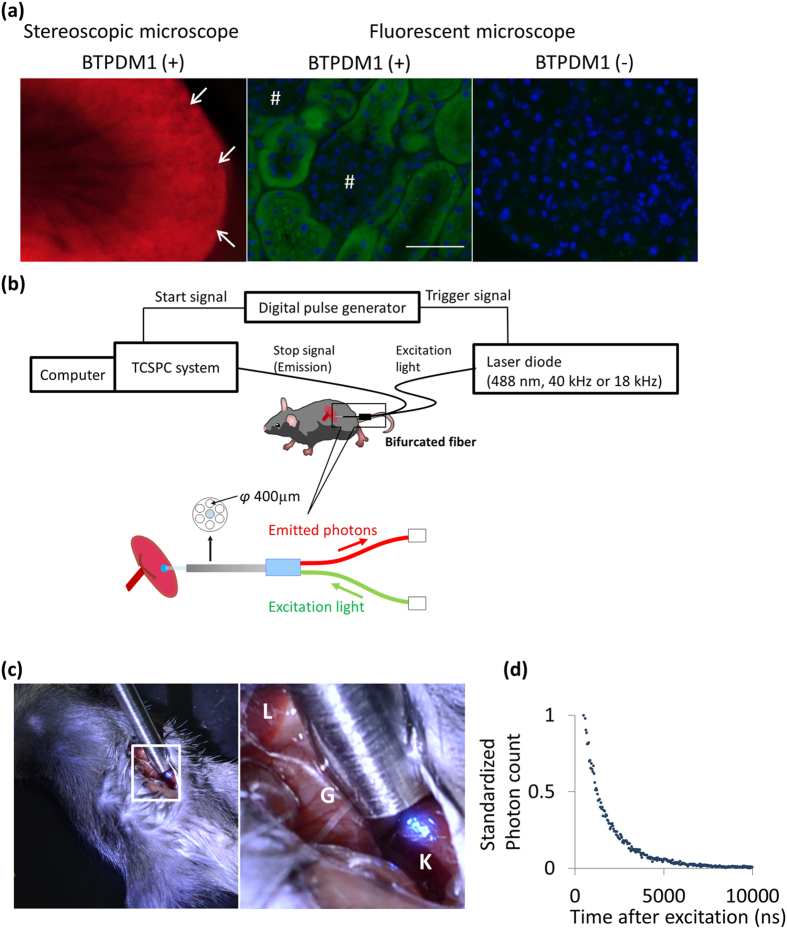
BTPDM1 distribution and phosphorescence lifetime measurements in normal mice. (**a**) Images of BTPDM1 distribution in kidney. The stereoscopic microscopic image is shown in the left panel. There were many dark circular areas in the cortex (arrow), which indicated BTPDM1 did not distribute in glomeruli. Phosphorescence images of kidney cortex with and without BTPDM1 obtained through fluorescence microscopy are shown in the center and right panels, respectively. Phosphorescent signals were seen in tubular, but not in glomerular areas (#). Green: BTPDM1, Blue: Hoechst 33258. Exposure time for BTPDM1: 0.5 s (center panel), 2 s (right panel). Original magnifications, 400×, scale bar: 50 μm. (**b**) Schematic diagram of the phosphorescence lifetime measurement system for living mouse. See Method for details. (**c**) Pictures of phosphorescence lifetime measurements of kidneys in a living mouse without black-out curtain. A magnified view of the left panel is shown in right panel. Liver (L), gut (G) and kidney (K) were seen. The excitation laser was spotted on the kidney and the diameter of the spotted area was approximately 3 mm. (**d**) An example of a phosphorescence decay curve from BTPDM1. Data acquisition time was about 60 s for 1024 channels. The maximum total photon counts were 1000. Photon count was standardized as the count at 500 ns to be one.

To assess whether BTPDM1 works as an intracellular oxygen concentration indicator *in vivo*, we investigated the distribution of BTPDM1 after intravenous administration. We observed cross-sections of kidney and frozen sections without any fixatives obtained from murine kidneys 30 min after BTPDM1 injection (Fig. 3a). Stereomicroscopic observations indicated that this phosphorescence dye did not distribute in glomerular areas, and observations of frozen sections revealed that BTPDM1 distributed inside tubular cells. This result is consistent with a previously reported result of inductively coupled plasma mass spectrometry (ICP-MS) indicating that BTPDM1 was distributed inside cells, and a minimal amount remained in the blood^14^. From these results we can conclude that the phosphorescence characteristics after BTPDM1 administration in the kidneys are affected almost entirely by the oxygen tension inside tubular cells, not in the blood stream. Next, we checked the decay curve of phosphorescence in the kidneys of living mice. The block diagram used to measure phosphorescence in murine kidney is shown in Fig. 3b. To measure the phosphorescence lifetime in the kidneys, mice were anesthetized and BTPDM1 (250 nmol in 25 μL dimethyl sulfoxide, diluted using PBS to 150 μL) was intravenously administered into the mice. Next, the abdomen was opened, and the gut was moved to expose the kidney. The surface temperature of the kidney was measured; it was always between 30 °C and 35 °C. The bifurcated fiber, which could irradiate the excitation light and detect the emitted photon, was then inserted near the kidney, and the excitation laser was irradiated. A representative picture of the measurement is shown in Fig. 3c. We could detect phosphorescence from the kidney, and the decay curve is shown in Fig. 3d.

We next investigated temporal changes of phosphorescence lifetime over time after BTPDM1 administration and found that phosphorescence lifetime remained stable up to 30 minutes (Supplementary Fig. 5). Thus, we measured the phosphorescence lifetime at 30 min after probe administration. To assess whether the phosphorescence lifetime was dependent on the probe concentration in the kidney tissues, phosphorescence lifetimes were measured after administration of two different probe amounts, 50 and 250 nmol, and the lifetime was verified to be independent of the probe amount under normal renal oxygen tension (Supplementary Fig. 6).

### Dependency of the phosphorescence lifetime on the tissue oxygen tension and extrapolation of the calibration curve

**Figure 4:**
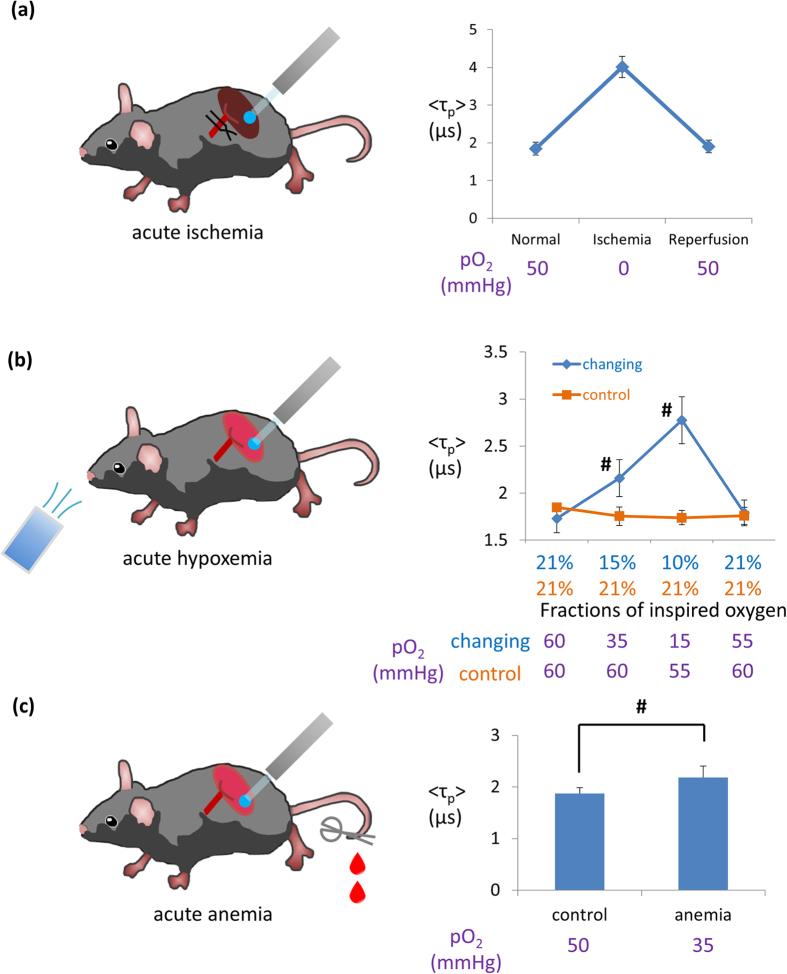
Phosphorescence lifetime change in hypoxic conditions of kidney. Experimental schemes (left panels) and phosphorescence lifetime alteration (right panels). The values written under graphs are converted pO_2_ (mmHg) from phosphorescence lifetimes by using the calibration line. (**a**) Phosphorescence lifetime alteration in acute ischemia/reperfusion. Acute ischemia and reperfusion were induced by clamping and declamping left renal artery and vein; n = 3. (**b**) Phosphorescence lifetime alterations in changing inspired oxygen concentration. The oxygen concentration of inhaling atmosphere was changed by digital gas mixing system; n = 4 (changing group), n = 3 (control group). (**c**) Phosphorescence lifetimes in acute anemia model. Anemia was induced by phlebotomy through the tail vein; n = 4 (control group), 6 (anemia group). In these three experiments, phosphorescence lifetimes actually elongated in hypoxic kidneys. Error bar: S.D. ^#^P < 0.05 by two-tailed unpaired t-test.

**Figure 5:**
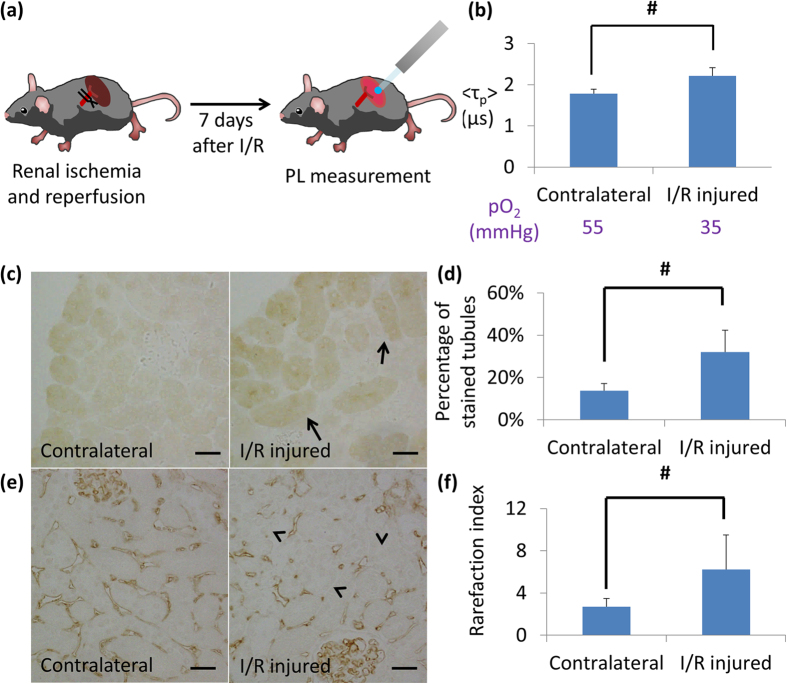
Phosphorescence lifetime change and proof of hypoxia in chronic damaged kidney. (**a**) Experimental scheme of unilateral ischemia-reperfusion model. (**b**) Average of phosphorescence lifetime of I/R injured and contralateral kidneys. Values under the graph show converted pO_2_ (mmHg) from phosphorescence lifetime by using the calibration line. n = 5. Error bar: S.D. ^#^P < 0.05 by two-tailed paired t-test. (**c,d**) Pimonidazole protein adducted immunohistochemistry of I/R injured model. Histological image of pimonidazole staining of I/R injured kidney (**c**, right panel) and contralateral kidney (**c**, left panel) are shown. More tubules in I/R injured kidneys are positive for pimonidazole adduct protein staining (representatives were indicated by arrows), which is supported by quantitative analysis of the proportion of stained tubules (**d**, n = 7). Original magnifications, 400×. Brightness and contrast were adjusted from original images. Scale bar: 30 μm. Error bar: S.D. ^#^P < 0.05 in two-tailed paired t-test. (**e,f**) Assessment of peritubular capillary (PTC) density using anti-CD31 antibody. Histological images of CD-31 staining of I/R injured kidney (**e**, right panel) and contralateral kidney (**e**, left panel) were shown. Disruption and distortion of PTC was seen (arrowhead). Quantitative analysis of rarefaction index shows the decrease in PTC in I/R injured kidney (**f**, n = 7). Original magnifications, 400×. Brightness and contrast were adjusted from original image. Scale bar: 30 μm. Error bar: S.D. ^#^P < 0.05 in two-tailed paired t-test.

To demonstrate the dependency of phosphorescence on tissue oxygen tension under pathological conditions, we conducted phosphorescence lifetime measurements in mice kidneys under three hypoxic conditions. We also attempted to convert phosphorescence lifetime to intracellular *p*O_2_ through the corresponding table in Supplementary Table 1 and affirmed the validity of the value of the intracellular oxygen concentrations we had obtained. Each converted intracellular oxygen tension is written under the graphs in Figs 4a–c and 5b.

The first experiment employed a model of acute renal ischemia. The phosphorescence lifetime immediately extended after clamping of the renal artery and restituted after the removal of the clamp (Fig. 4a), showing that this phosphorescence probe worked as a real-time indicator of severe hypoxia by measuring phosphorescence lifetimes *in vivo*. We also found that the change in phosphorescence lifetime occurred just after clamping and de-clamping (Supplementary Fig. 7). This indicated that our phosphorescence lifetime measurement technique quickly reflected changes in oxygen tension in the renal tissue. The second experiment involved mice inhaling lower oxygen concentration, and our probe and technique allowed the detection of hypoxia induced by inspiring 15% oxygen (Fig. 4b). This experiment also substantiated the reversibility of the phosphorescence lifetime. The last experiment involved phosphorescence lifetime measurements in anemic mice. Anemia was induced through phlebotomy and the extent of anemia is shown in Supplementary Fig. 8. In these mice, the phosphorescence lifetime in the kidneys was longer than that in control mice (Fig. 4c).

### Oxygen tension of chronic damaged kidney from ischemia-reperfusion

We next attempted to assess the renal oxygen tension in a model of chronic kidney disease (CKD) by measuring phosphorescence lifetimes. In this case, we adopted a unilateral ischemia-reperfusion (I/R) model, since there was little fibrosis 7 days after I/R, followed by development of severe fibrosis 4 weeks after I/R. Thus, we examined the kidney at 7 days after I/R and confirmed no significant fibrosis in the I/R-injured kidneys (Fig. 5a and Supplementary Fig. 9a). We checked BTPDM1 distribution in the I/R injured and contralateral kidneys and confirmed that BTPDM1 also distributes mainly in the tubules of the I/R-injured mice, although the probe concentrations in the tubules were thought to be lower than normal kidney based on weaker phosphorescence (Supplementary Fig. 9b). This identification of probe distribution was important since the phosphorescence lifetime might be dependent on cell types, even under the same O_2_ concentrations. Next, the phosphorescence decay curve was measured in the I/R-injured and contralateral kidneys in the same individuals. There was a significant difference in phosphorescence lifetime in this I/R-injured model (Fig. 5b). Together with the same distribution of BTPDM1, these differences between phosphorescence lifetimes should represent the differences between oxygen tension in tubular cells. In addition to detecting hypoxia in the I/R-injured kidneys, we evaluated hypoxia quantitatively by using the Supplementary table 1, and found that the phosphorescence lifetime in I/R-injured kidneys corresponded to that in HK-2 cells incubated at 35 mmHg of oxygen (4.6% O_2_), while the phosphorescence lifetime in contralateral kidneys corresponded to 55 mmHg of oxygen (7.2% O_2_).

To affirm that the I/R-injured kidneys were more hypoxic than contralateral kidneys, we performed pimonidazole and CD-31 immunohistochemistry. Hematoxylin–eosin staining and negative control of the immunohistochemistry under the same magnification are shown in Supplementary Fig. 10. The fraction of stained tubules in cortical areas was more in I/R-injured kidneys by pimonidazole protein adduct immunohistochemistry (Fig. 5c,d), and it confirmed that the I/R-injured kidneys were more hypoxic than contralateral kidneys. The rarefaction of peritubular capillaries was revealed in the I/R-injured kidneys through immunohistochemistry using CD-31 (Fig. 5e,f). This was consistent with hypoxia in the I/R-injured kidneys because renal hypoxia observed in CKD was closely associated with the loss of density of peritubular capillaries^4^,^15^,^16^.

## Discussion

In this study, we demonstrated the highly sensitive, rapid, and quantitative evaluation of intracellular oxygen tension via phosphorescence lifetime measurements using BTPDM1. This technique can be applied to other organs such as liver^17^ and heart^18^ in which hypoxia plays a major role in pathological processes. This technique should be also useful in researching the pancreas because oxygen tension affects cell viability in the pancreas^19^.

It is worth mentioning that intra-tissue distribution of our probe, BTPDM1, is different from that of other phosphorescence probes used *in vivo* such as PtP-C343, and Oxyphors G2 and G4; these previously reported phosphorescence probes distribute mainly in extracellular fluid such as blood or interstitial fluid, while our probe exhibited intracellular distribution in murine kidneys. Accordingly, we generated a calibration curve under appropriate conditions for our purpose.

We found phosphorescence lifetimes differed between cultured cells and DMPC membranes as well as between cultured cell types. The differences may be explained by factors such as cell-type dependent consumption of oxygen in mitochondria^20^. Irrespective of the case, we reasoned that tissue specific calibration curves should be prepared for intracellular oxygen tension for each organ. This can be a limitation of this technique. Since organ specific calibrations are needed, direct comparison of oxygen tension between different organs is difficult. Thus, instead of using pH buffered solution with or without bovine serum albumin to mimic the extracellular fluid used for extracellularly-distributed probes^8^,^21^,^22^ or using cancer cells to mimic intra-tumor conditions used for tumor imaging with BTPDM1^14^, we used a strategy to generate a calibration curve in cultured proximal tubular cells, which mimics intracellular conditions in murine kidneys *in vivo.*

We validated our methodology through comparisons of the *p*O_2_ obtained in our experiments to those previously reported in which oxygen electrodes were used^23^,^24^,^25^,^26^,^27^. Looking comprehensively at the three experiments in Fig. 3a–c, the phosphorescence lifetime of normal kidneys was thought to be 1.7–1.9 μs and they were converted to 50–60 mmHg (6.6%–7.9% O_2_). These values were almost equivalent to the values obtained by oxygen electrodes, which were reported to be 45‒50 mmHg (5.9%–6.6% O_2_) in rat kidney cortex^23^,^24^,^25^,^26^. A previous report using oxygen electrodes revealed *p*O_2_ was approximately 35 mmHg (4.6% O_2_) in kidneys under a 10% oxygen concentration (76 mmHg)^27^. In contrast, we reported *p*O_2_ in kidneys was around 15 mmHg (2.0% O_2_) under a 10% oxygen concentration (Fig. 3b). This discrepancy may be explained by the differences in what we actually measured. Our method measured intracellular oxygen tension whereas an oxygen electrode measures oxygen tension in the microcirculation. It should be also mentioned that our calibration line cannot be applied to oxygen tensions <10 mmHg (1.2% oxygen [Fig. 2c]). The standard level of oxygen tension in the renal cortex was reported to be 45–50 mmHg as previously mentioned, and all of pO_2_ *in vivo* discussed in this study was >15 mmHg (2.0% O_2_). Thus, our calibration line can be applied to changes in oxygen tensions under both physiological and pathophysiological conditions. In addition, the phosphorescence lifetime in RPTEC was equivalent to those in HK-2 cells under the same physiological and pathophysiological oxygen tensions, which supports the application of the calibration line obtained from HK-2 cells to phosphorescence lifetime measurements *in vivo*.

Measurement of oxygen tension in more fibrotic kidneys is a subject of future studies. Our preliminary studies suggested that BTPDM1 concentrations in fibrotic kidneys would be much less than those in pre-fibrotic normal kidneys. Since we adopted a time-correlated single photon counting (TCSPC) system, which needs to accumulate phosphorescence signals, a certain probe concentration is needed; the lower probe concentration hinders phosphorescence lifetime measurements. This problem can be solved through future improvements in the detection sensitivity.

We demonstrated that phosphorescence lifetime measurement using BTPDM1 was a quantitative and reliable technique for detecting and assessing intracellular hypoxia in kidneys. This time we measured the phosphorescence lifetime of a cell population in renal cortical tubules, thus the measured phosphorescence lifetime was thought to be the average of each phosphorescence lifetime of a single cell. Our technique makes the estimations of intracellular *p*O_2_ *in vivo* feasible. Since intracellular oxygen tension is directly related to various cellular hypoxic reactions, this novel method will shed new light on research of oxygen biology *in vivo*.

## Method

### Cell experiment

HK-2 cells were incubated at 37 °C in a mixture of Dulbecco’s Modified Eagle’s Medium (DMEM) and Ham’s F-12 Nutrient Mixture containing 10% Fetal Bovine Serum (FBS) and 100 U/ml penicillin and 100 μg/ml streptomycin. Cells were grown in humidified 5%-CO_2_ enriched atmosphere at 37 °C. For RPTEC (CC-2553, Lonza Ltd., Basel, Switzerland), cells were incubated in REGM (CC-3190, Lonza Ltd., Basel, Switzerland).

For phosphorescence imaging or phosphorescence lifetime measurements in HK-2 cells, all cells were cultured on glass base dishes, and the media was changed to DMEM/F-12 without phenol red or FBS; BTPDM1 was added at a final concentration of 500 nM. After 30 min incubation, the media was again changed to FBS- and BTPDM1-free media, followed by phosphorescence imaging or phosphorescence lifetime measurements. For RPTEC, the media was REGM and BTPDM1 was added in the same amount 30 min before phosphorescence lifetime measurements, and the media was changed to new REGM without BTPDM1 for phosphorescence lifetime measurements.

In order to merge LysoTracker signals with BTPDM1, cells were washed using HBSS after BTPDM1 administration, and LysoTracker DND-99 (L-7528, Life technologies, Carlsbad, CA) was added at a final concentration of 50 nM, washed 3×, and observed under the microscope. The characteristics of the excitation filter, dichroic mirror, and emission filter used to detect BTPDM1 are shown in Supplementary Fig. 11. To assess the toxicity of BTPDM1, HK-2 cells were incubated for 24 h with various concentrations of BTPDM1, and cell viability was assessed using a Cell Counting Kit-8 (CK04, Dojindo Laboratories, Kumamoto, Japan).

### Animal Experiments

All protocols for animal experiments were approved by the Ethical Committee on Animal Experiments of the University of Tokyo (M-P13-040) and Gumma University (13-031), and all animal experiments were conducted in accordance with the institutional guidelines.

Eight-week-old C57BL/6J male mice were used in this study. For surgery and phosphorescence lifetime measurements in mice, general anesthesia was administered via 40 mg/kg intraperitoneal injection of pentobarbital, and 8 mg/kg pentobarbital was added if necessary. For phosphorescence lifetime measurements, all of the experiments were done with mice on a heater mat, and BTPDM1 was intravenously injected 30 min before phosphorescence lifetime measurements. Under general anesthesia, the abdomen was opened via median incision, and kidneys were exposed after the guts were moved. We measured the surface temperature of the kidneys using a thermometer (TSU-0125, Tokai Hit Co., Ltd, Shizuoka, Japan) and then the kidney was irradiated with the excitation laser. For acute ischemia/reperfusion, the abdomen was opened and the left renal pedicle was exposed and ligated through a suture. After 20–30 min, the suture was cut; the time variation depended on depth of anesthesia. If anesthesia was weak, we added pentobarbital and waited. The color of the kidneys was carefully monitored as to whether enough ischemia had been induced and released. As for the chronic ischemia/reperfusion model, ischemia and reperfusion were performed in a similar manner to acute model. The differences, however, were that ischemia was induced by the clip and that ischemic time was 30 min for chronic ischemia/reperfusion model. Seven days after ischemia/reperfusion, phosphorescence lifetimes or histological changes were evaluated. For measuring phosphorescence lifetimes under various oxygen concentrations, mice inhaled room air (21% oxygen) or a mixture of air and N_2_ using a digital gas mixing system GM-8000 (Tokai-Hit Co., Ltd. Shizuoka, Japan). In this experiment, the control group was given the same amount of time to always inhale room air. The anemia model was induced by phlebotomy of 300 μl blood over 2 consecutive days, and on the day after completion of phlebotomy, phosphorescence lifetimes or hematocrits were measured. For measuring hematocrits, blood was absorbed to heparinized capillary tubes designed for hematocrit measurements (Terumo Co. Ltd, Tokyo, Japan), and centrifuged for 5 min at 12,000 rpm.

### Stereoscopic microscopy

Kidneys were placed on a stereoscopic microscope (Leica M165 FC, Leica Microsystems, Heerbrugg, Switzerland) just after being resected and halved to observe the distribution of BTPDM1. Fluorescence was observed using an excitation filter of 470/20 and an emission filter of 510LP.

### Frozen sections

To prepare frozen sections after euthanasia mice kidneys were immediately frozen with O.C.T. compound (Sakura Finetek Japan Co., Ltd., Tokyo, Japan) without any fixation to avoid efflux of the probe. Kidney sections of 4 μm thickness were observed under the fluorescence microscope BZ-710 (Keyence Corporation, Osaka, Japan) after counterstaining of nuclei using Hoechst 33258.

### Immunohistochemistry

Histological analysis was performed via optical microscopy of formalin-fixed, paraffin embedded sections of 3 μm thickness with periodic acid–Schiff reagent (PAS) and Trichrome–Masson staining (MT). For immunohistochemistry, the hypoxyprobe in Hypoxyprobe-Omni Kit (HP3-100, Hypoxyprobe, Inc., Burlington, MA) was administered via peritoneal injection 120 min before euthanasia. After euthanasia, kidneys were fixed using formalin or Carnoy’s solution and embedded in paraffin. Kidney sections of 3 μm thickness were incubated in Hypoxyprobe-Omni antibody (1:200; HP3-100, Hypoxyprobe, Inc., Burlington, MA) or CD-31 antibody (1:20; DIA310, Dianova, Hamburg, Germany) overnight at 4 °C, biotinylated goat anti-rabbit IgG antibody (1:1000; BA-1000, Vector Laboratories, Inc., Burlingame, CA) or biotinylated goat anti-rat IgG antibody (1:400; BA-9400, Vector Laboratories, Inc., Burlingame, CA) for 40 min and HRP-conjugated avidin D (1:2000; A-2004, Vector Laboratories, Burlingame, CA) for 30 min. Color was developed using diaminobenzidine (045-22833, Wako Pure Chemical Industries, Ltd., Osaka, Japan) and H_2_O_2_ at 37 °C.

For quantitative analysis of pimonidazole protein adduct immunohistochemistry, the number of stained and total tubules was counted in five high magnification fields (400×) for each mouse, and the total percentage of stained tubules was evaluated. Density of peritubular capillaries was evaluated using a rarefaction index, as previously described^28^. In this study, we used 10 × 10 grids under 400× magnification, squares without CD31-positive capillaries were counted, and the number in five fields was averaged.

### Phosphorescence Lifetime Measurements

BTPDM1 synthesis and phosphorescence lifetime measurements were performed according to our previous report^14^. For incubated cells, phosphorescence lifetime was measured using an inverted microscope (IX71, Olympus, Tokyo, Japan) with an O_2_ concentration-changeable temperature feedback heating stage top incubator (Inub-Onics-F1-H2, Gm-8000, Tokai Hit Co., Ltd, Shizuoka, Japan) and a laser diode (iBeam smart-S 488-S: 488 nm; pulse width, 20 ns; repetition rate, 40 kHz or 18 kHz, Toptica Photonics AG, Gräfelfing, Germany) as the excitation light source and a time-correlated single photon counting (TCSPC) system (Quantaurus-Tau C11367, Hamamatsu Photonics K.K., Shizuoka, Japan). Phosphorescence lifetime measurements were performed with 70% confluent cells in 250 μl of medium. To make a calibration curve, phosphorescence lifetime was measured 1 h after changing oxygen concentrations of the incubation medium. Phosphorescence lifetime measurements were made 4×, and the average lifetime was taken as 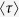.

The *in vivo* phosphorescence lifetime of BTPDM1 in kidneys was measured using the same lifetime measurement system combined with a seven-way branched fiber that was used to irradiate the kidney surface (about 3 mm diameter) of a mouse and collect the emission from the irradiated area. Of the seven bifurcations, the surrounding six were used to irradiate the kidney, and the emission from the kidney passed the other one. During measurements, mice and the fiber were under black-out conditions.

All of the decay curves obtained from incubated cells and kidneys could be fitted bi-exponentially. As for the calculated average lifetime, we adopted an intensity-averaged lifetime 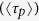, which is expressed in the equation below, where A_1_ and A_2_ are the pre-exponential factors of each component and τ_1_ and τ_2_ are the lifetimes of each.


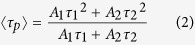


All of the measurements of phosphorescence lifetimes were repeated 4×, and 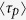 was adopted to be the lifetime of each condition, unless there were obvious errors.

### Statistical Analyses

All data are presented as means ± standard deviation. Statistical comparisons between two groups were made using paired or unpaired student’s t-test.

## Additional Information

**How to cite this article**: Hirakawa, Y. *et al.* Quantitating intracellular oxygen tension *in vivo* by phosphorescence lifetime measurement. *Sci. Rep.* **5**, 17838; doi: 10.1038/srep17838 (2015).

## Supplementary Material

Supplementary Information
