# Supplementary material for: Quantitating intracellular oxygen tension in vivo by phosphorescence lifetime measurement
Source: Sci Rep. 2015 Dec 8;5:17838. doi: 10.1038/srep17838 (PMC4672317; doi:10.1038/srep17838)
Supplement: Supplementary Information [file srep17838-s1.pdf]

Quantitating intracellular oxygen tension *in vivo* by phosphorescence lifetime measurement.

Yosuke Hirakawa, Toshitada Yoshihara, Mako Kamiya, Imari Mimura, Daichi Fujikura, Tsuyoshi Masuda, Ryohei Kikuchi, Ippei Takahashi, Yasuteru Urano, Seiji Tobita, Masaomi Nangaku

Supplementary Figure legends

Supplementary Figure 1. Structure and absorption/phosphorescence spectrum of BTPDM1

(a) Chemical structure of BTPDM1. (b) absorption (blue) and phosphorescence (red) spectrum of BTPDM1.

Supplementary Figure 2. Phosphorescence lifetime in various oxygen concentrations in HK-2 cells at 30 °C

Phosphorescence lifetime of BTPDM1 in HK-2 cells was measured under various pO_2 at 30 °C, and the reciprocal plots of phosphorescence lifetime and linear approximation were made. Approximation formula and coefficient of determination was also shown. As is the same to Figure 2c, phosphorescence lifetime when pO_2 is zero was far away from the approximate line obtained from the other plots, thus it was ignored when drawing calibration line and determining the constants. Error bar: S.D.

Supplementary Figure 3. Phosphorescence lifetime in RPTEC

Phosphorescence lifetime of BTPDM1 in RPTEC was measured under various pO_2 at 37 °C, and the reciprocal plots of phosphorescence lifetime were shown. Each phosphorescence lifetime was the average of four measurements. The lifetime of HK-2 and the calibration line in HK-2 were also shown (data shown in Figure 2c). Error bar:

S.D.

Supplementary Figure 4. Toxicity assay of BTPDM1 on HK-2 cells

The number of viable cells was counted 2 h after BTPDM1 administration. LC50 was assumed to be around 5 μM, and we used BTPDM1 at lower concentrations in other cellular experiments. Error bar: S.D.

Supplementary Figure 5. Time course of phosphorescence lifetime after BTPDM1 administration

Phosphorescence lifetimes were serially measured in kidneys after BTPDM1 administration in a single mouse. Phosphorescence lifetimes were much longer immediately after BTPDM1 injection and the lifetime was stable at around 30 min after probe injection. The probable reason for a longer lifetime immediately after administration of the probe was the interactions with serum proteins, such as albumin according to its cationic charge.

Supplementary Figure 6. Dose dependency of phosphorescence lifetime after administration of BTPDM1

Phosphorescence lifetime of the mice kidney after administration of two different amounts of BTPDM1. There were no significant differences between the two groups. n = 3 for each. Error bar: S.D.

Supplementary Figure 7. Variation of phosphorescence lifetime by acute ischemia and reperfusion

The change in phosphorescence lifetimes over time after ligation of renal vessels (a) and their opening (b). Phosphorescence lifetime changed drastically and quickly after ligation, and elongated slowly for around 15 minutes. After reperfusion, the phosphorescence lifetime also rapidly changed, however it did not change over time.

Supplementary Figure 8. Hematocrit of anemia model and control mice

Hematocrit levels of anemic and control groups. Hematocrits were measured after 300 µl blood discard in two consecutive days. The value of hematocrits were shown as mean ± S.D. n = 5 for each group. Error bar: S.D. #: $P < 0.05$ by one-tailed unpaired t-test.

Supplementary Figure 9. Histological images and BTPDM1 distribution of unilateral I/R injury model

(a) Light microscope images. Periodic acid–Schiff reagent (PAS) staining of I/R injured (right panel) and contralateral kidney (left panel) are shown in upper images, and Trichrome–Masson (MT) staining of I/R injured (right panel) and contralateral kidney (left panel) in lower images. Original magnification, 100×. Scale bar: 100 µm (b) Fluorescence microscope images. I/R injured kidney with BTPDM1 (upper) and without BTPDM1 (lower) are shown in right images, and contralateral kidney with BTPDM1

(upper) and without BTPDM1 (lower) are in left panels. Exposure time for BTPDM1: 3s (I/R injured) and 1s (contralateral). Original magnification, 400×. Scale bar: 50 μm

Supplementary Figure 10. Staining control for immunohistochemistry of I/R model

(a) Hematoxylin–eosin staining of I/R injured kidney (right panel) and contralateral kidney (left panel) of I/R injured model. Original magnification, 400×. Scale bar: 20 μm. (b) Negative control staining for pimonidazole staining and CD31 staining. Original magnification, 400×. Brightness and contrast were adjusted from original images. Scale bar: 30 μm. Error bar: S.D.

Supplementary Figure 11. Excitation and emission filters to detect BTPDM1 phosphorescence

The transmission rates of excitation filter, dichroic mirror and emission filter are shown in blue, red, and green, respectively.

Supplementary Table legend

Supplementary Table 1 Correspondence table between oxygen tension and phosphorescence lifetime in HK-2 cells

Partial pressure of oxygen was estimated from phosphorescence lifetimes by using the calibration line obtained in Fig. 2c and Supplementary Fig. 2. The partial pressure of oxygen was described at 5 mmHg interval according to our detection sensitivity. There

were no significant differences in estimated oxygen tension between 37 °C and 30 °C in these significant digits.

Supplementary Figure 1 (Nangaku)

(a)

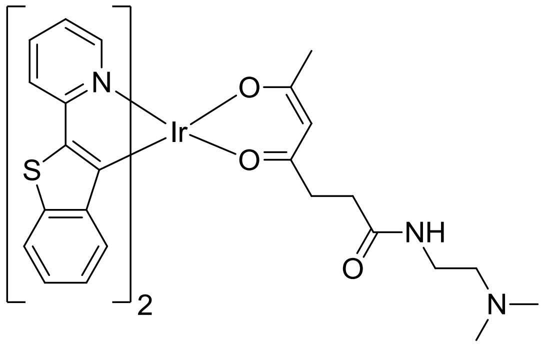

(b)

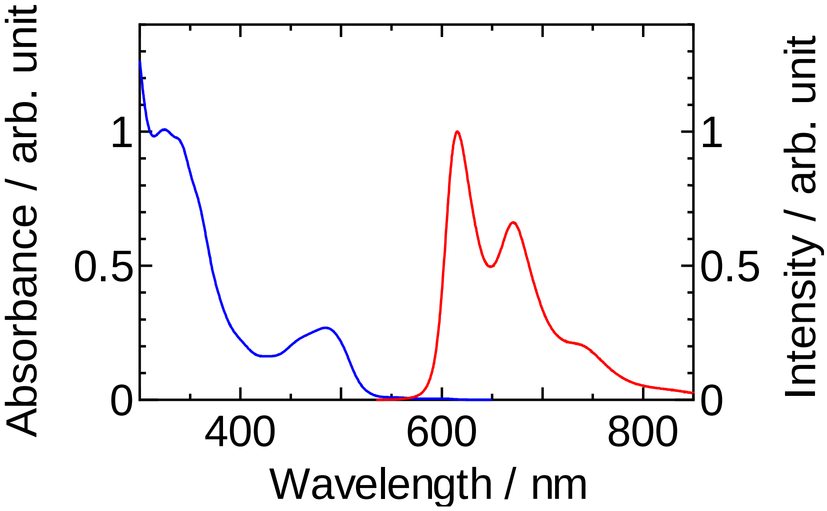

Supplementary Figure 2 (Nangaku)

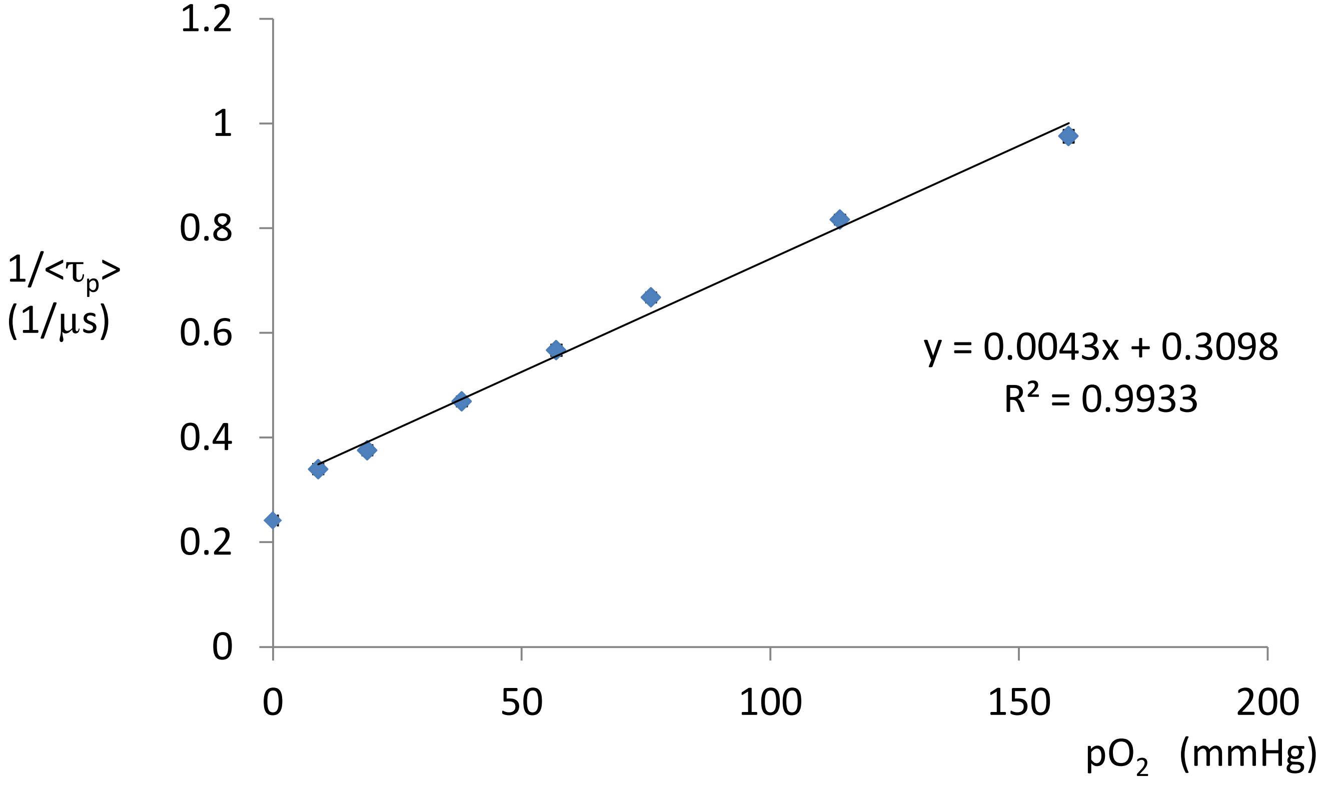

Supplementary Figure 3 (Nangaku)

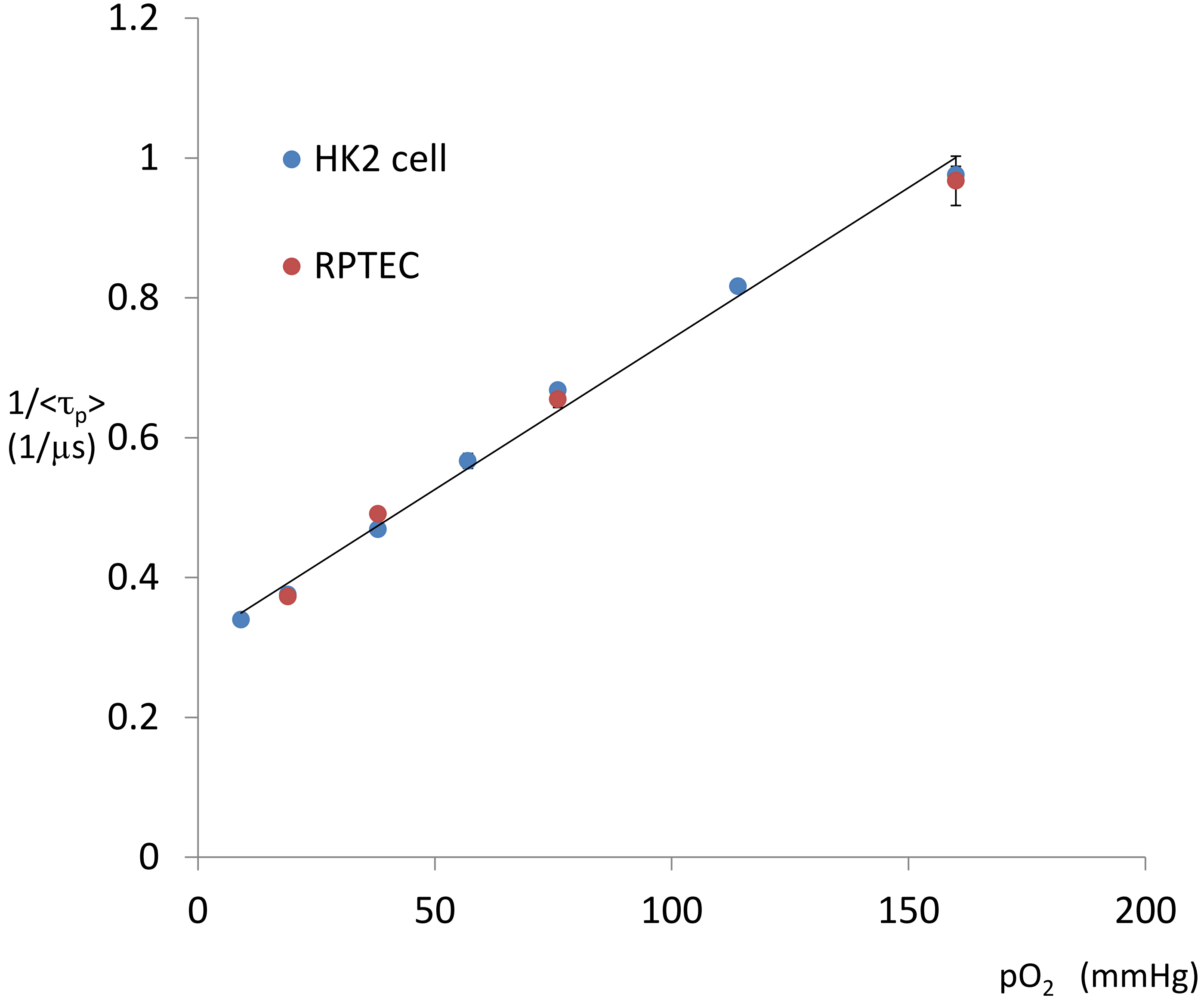

Supplementary Figure 4 (Nangaku)

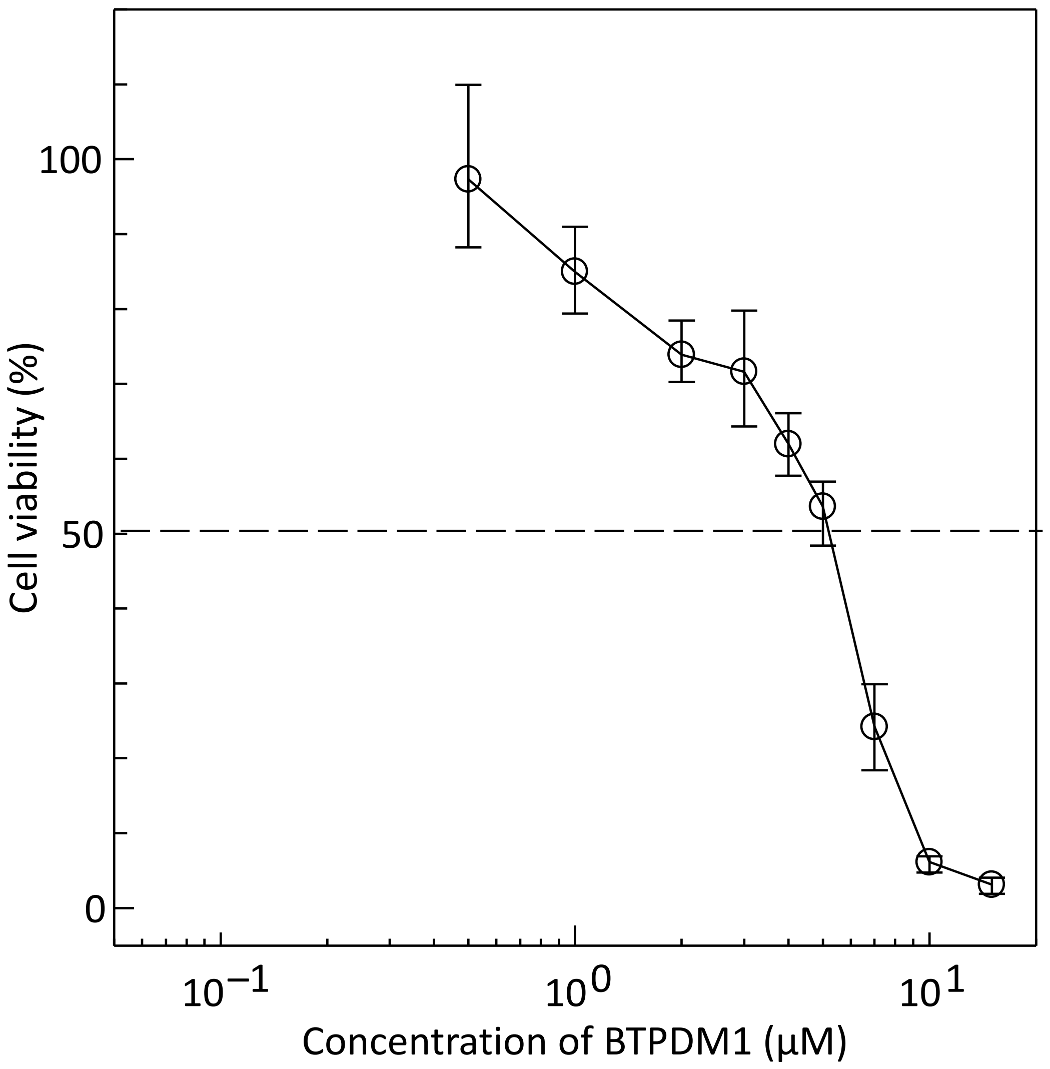

Supplementary Figure 5 (Nangaku)

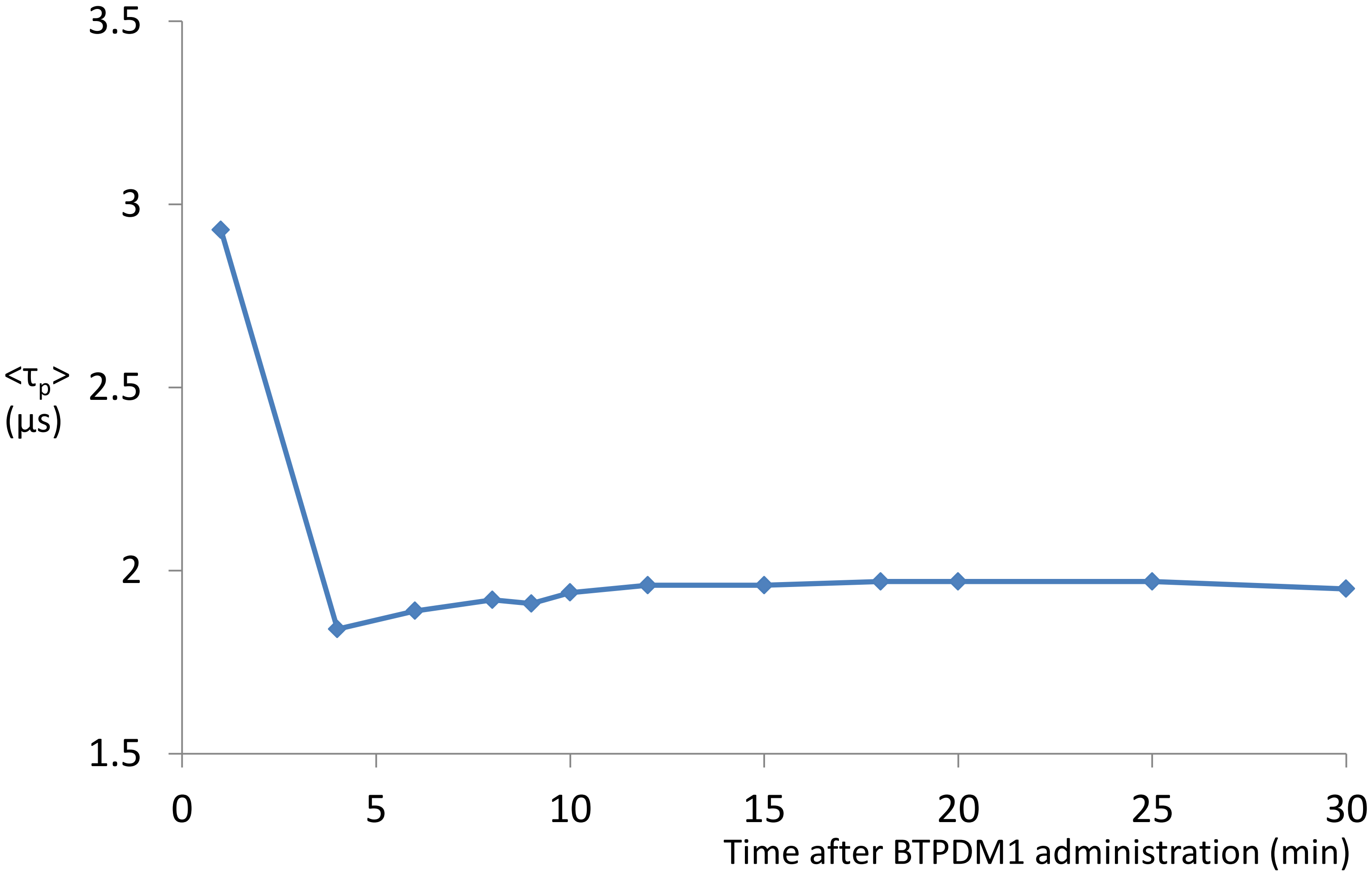

Supplementary Figure 6 (Nangaku)

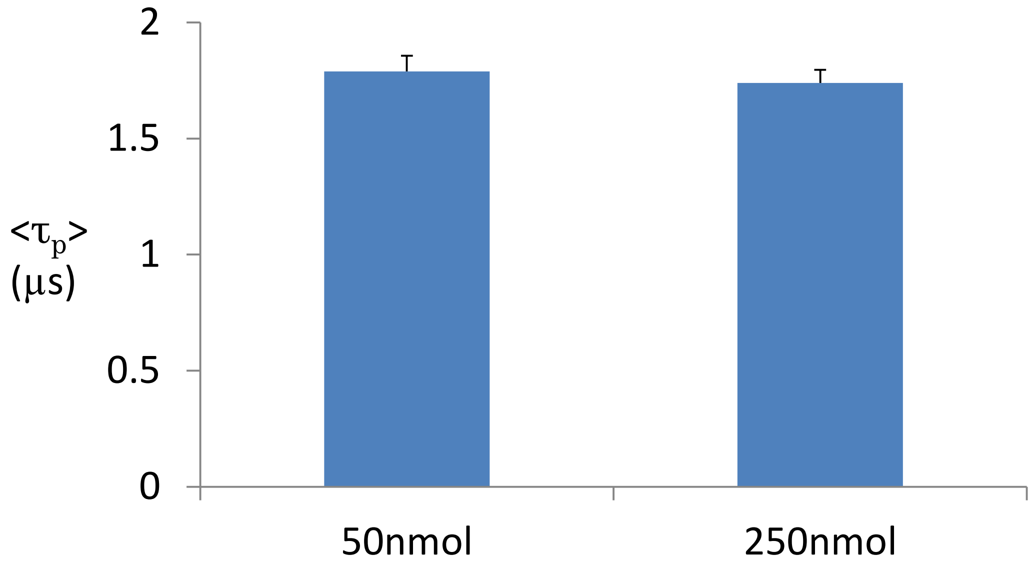

Supplementary Figure 7 (Nangaku)

(a)

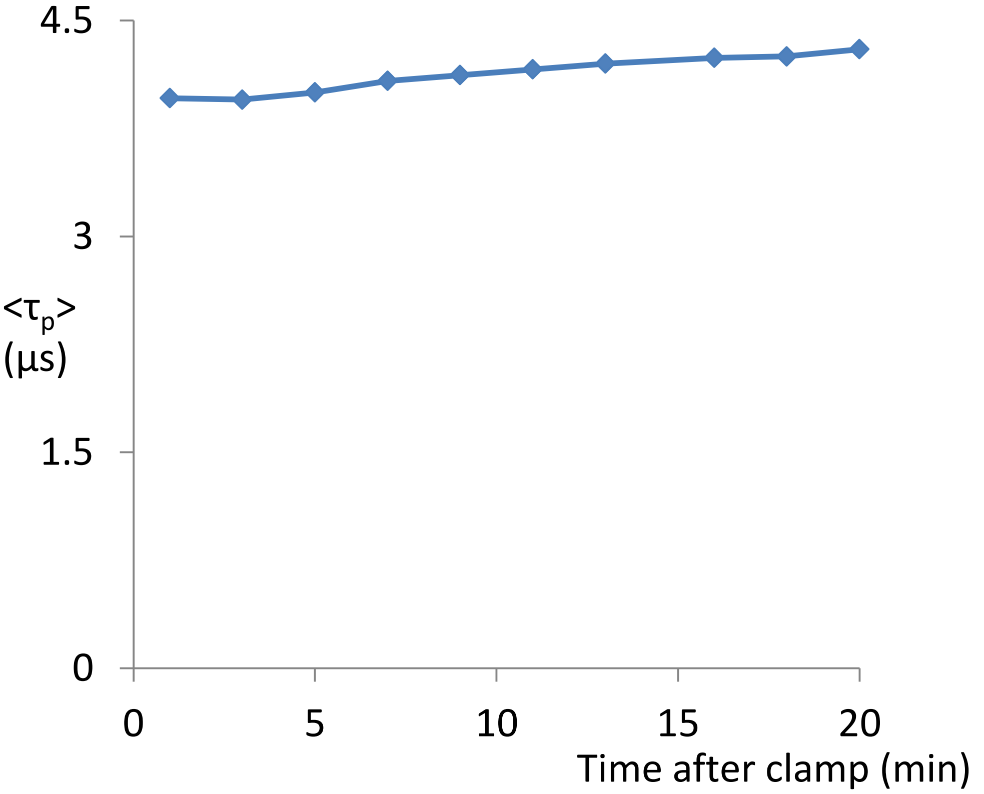

(b)

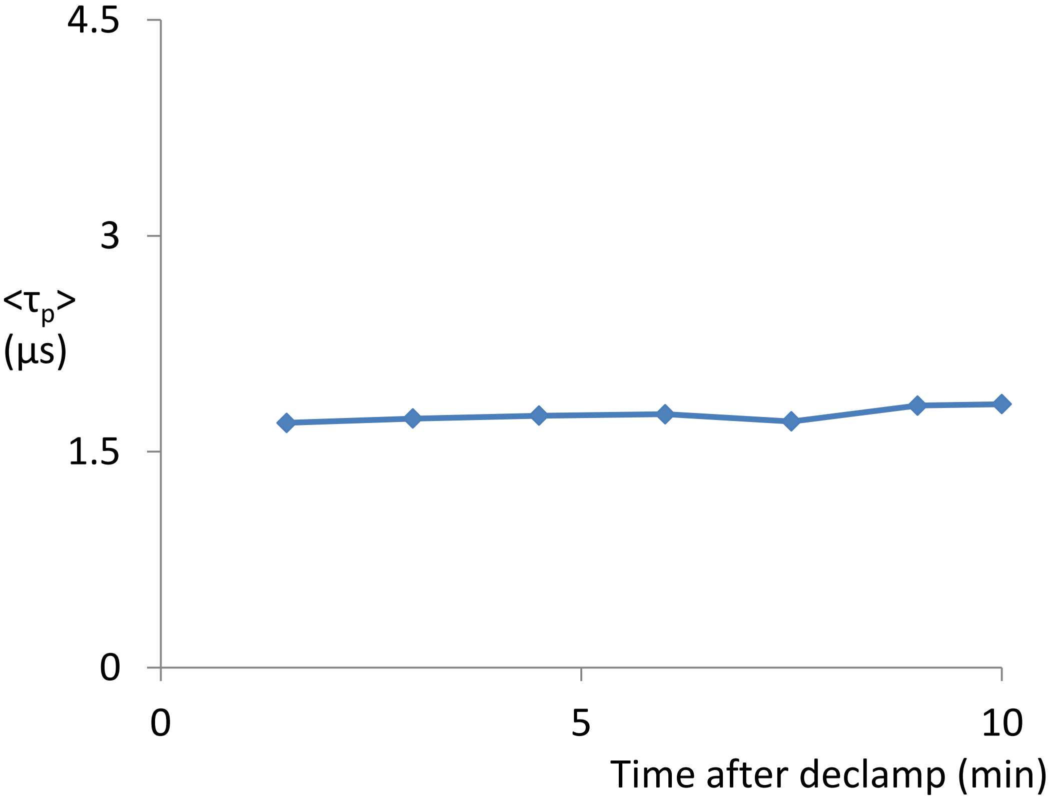

Supplementary Figure 8 (Nangaku)

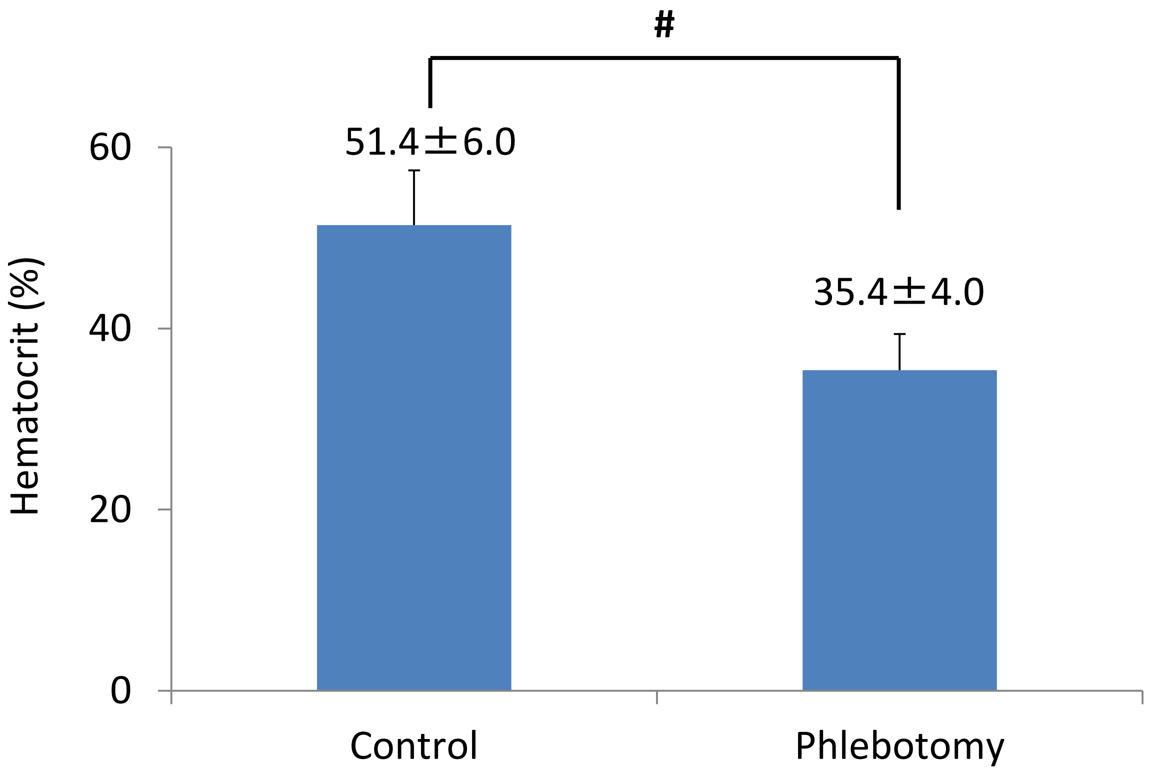

Supplementary Figure 9 (Nangaku)

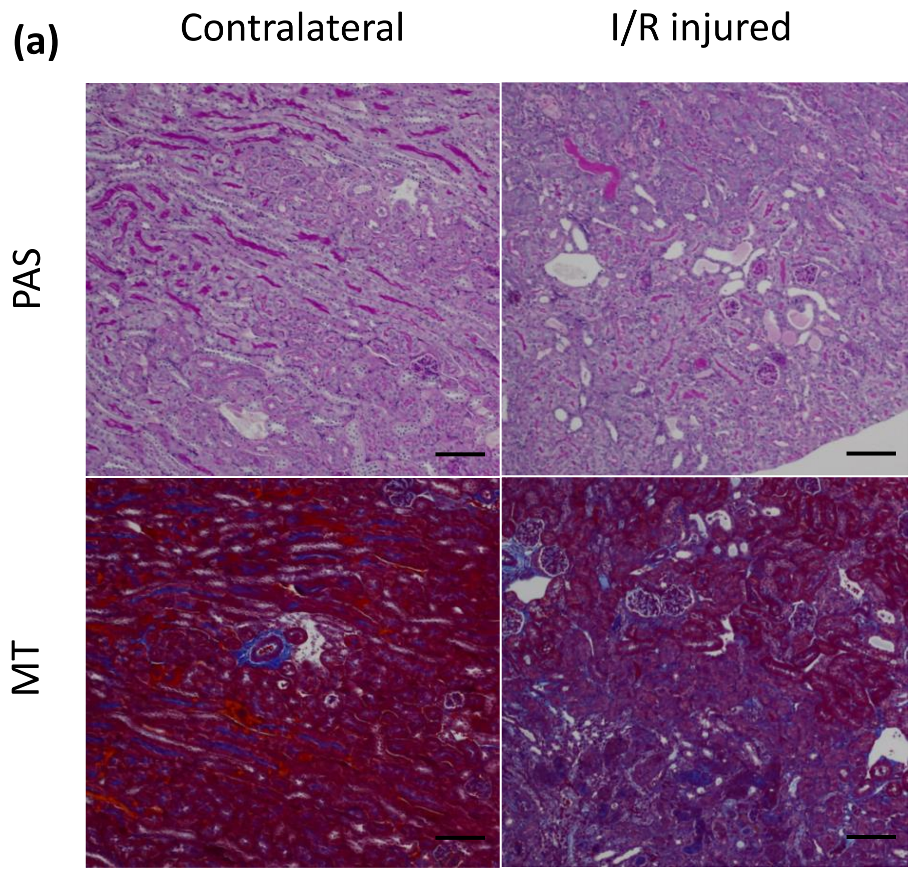

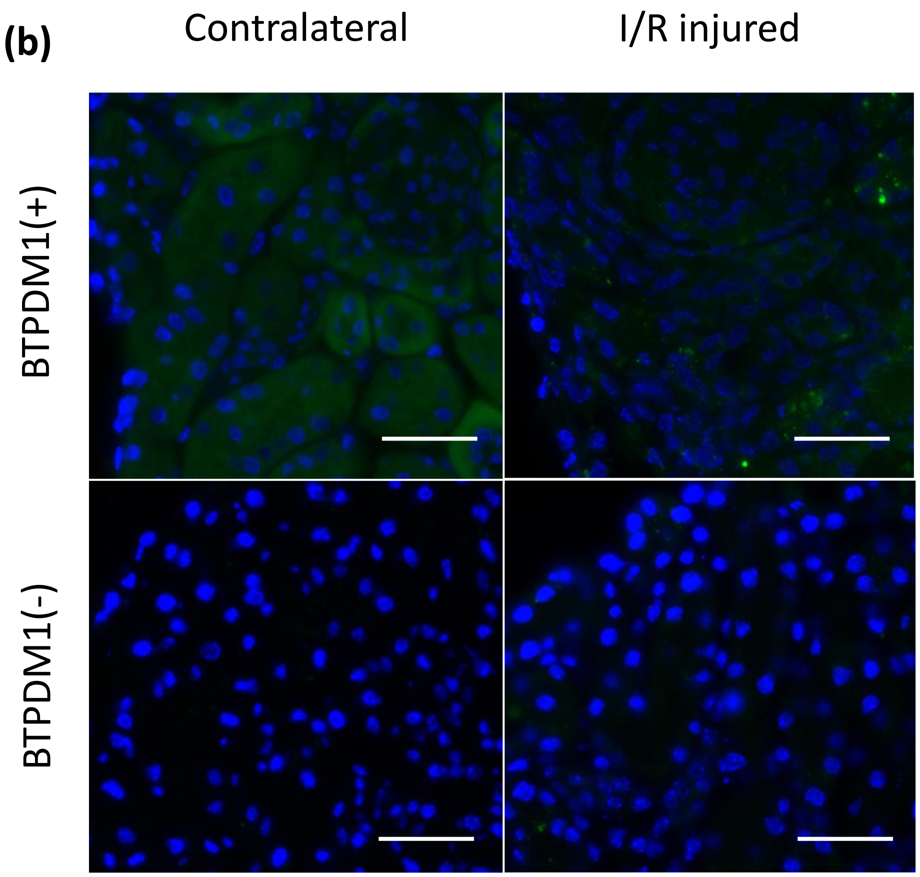

Supplementary Figure 10 (Nangaku)

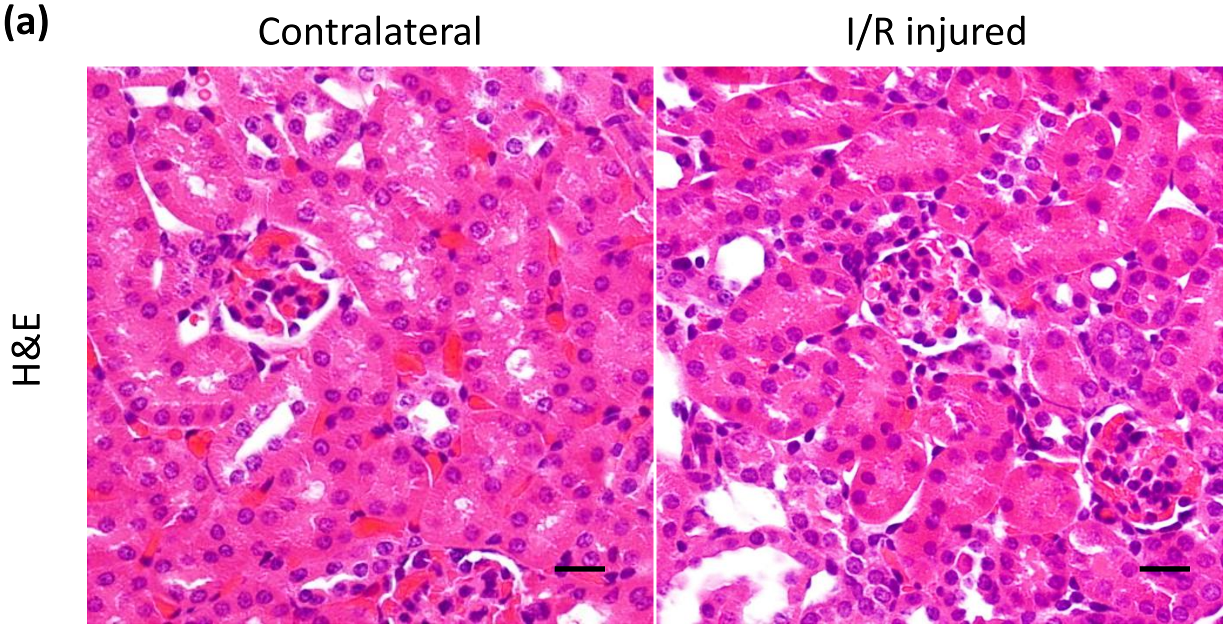

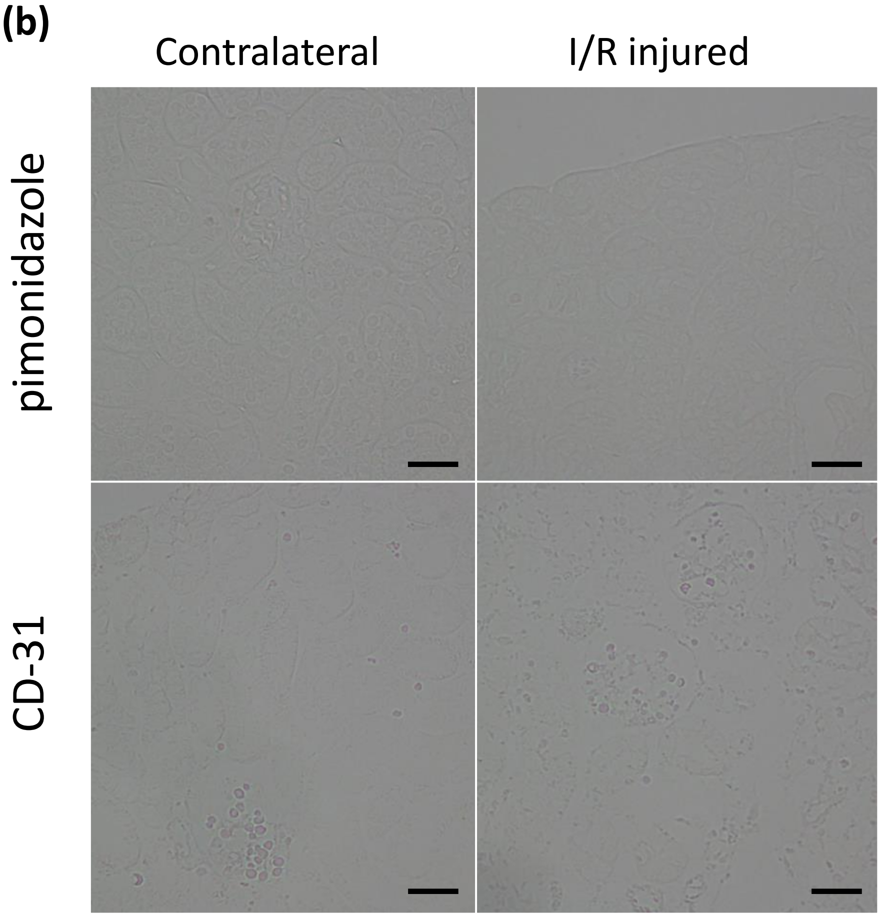

Supplementary Figure 11 (Nangaku)

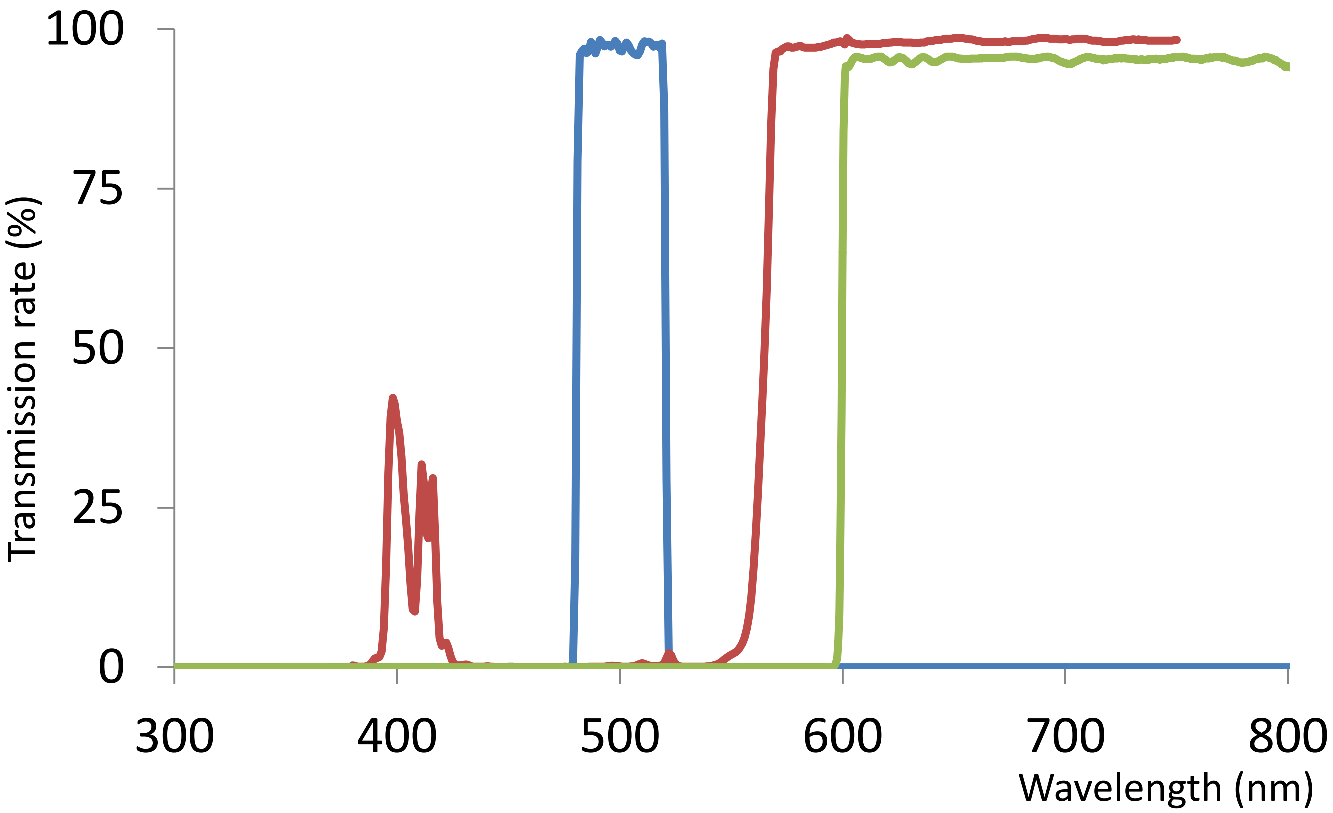

Supplementary Table 1 (Nangaku)

37˚C		30˚C	
$<\tau_p>$ (μs)	pO_2(mmHg)	$<\tau_p>$ (μs)	pO_2(mmHg)
4.0	0	4.1	0
2.9	10	2.9	10
2.8–2.7	15	2.8–2.6	15
2.6	20	2.5	20
2.5–2.4	25	2.4	25
2.3	30	2.3	30
2.2	35	2.2	35
2.1	40	2.1	40
2.0	45	2.0	45
1.9	50	1.9	50
1.8	55	1.8	55
1.7	60	1.7	65
1.6	70	1.6	75
1.5	75	1.5	85
